# Unambiguous molecular detections with multiple genetic approach for the complicated chromosome 22q11 deletion syndrome

**DOI:** 10.1186/1471-2350-10-16

**Published:** 2009-02-25

**Authors:** Chen Yang, Cheng-Hung Huang, Mei-Leng Cheong, Kun-Long Hung, Lung-Huang Lin, Yeong-Seng Yu, Chih-Cheng Chien, Huei-Chen Huang, Chan-Wei Chen, Chi-Jung Huang

**Affiliations:** 1 Division of Genetics, Department of Pediatrics, Taipei Medical University Hospital, Taipei 11031, Taiwan; 2 Department of Pediatrics, Cathay General Hospital Sijhih, Taipei 22174, Taiwan; 3 Graduate Insitute of Clinical Medicine, School of Medicine, Taipei Medical University, Taipei 11031, Taiwan; 4 Department of Gynecology and Obstetrics, Cathay General Hospital, Taipei 10630, Taiwan; 5 Department of Pediatrics, Cathay General Hospital, Taipei 10630, Taiwan; 6 School of Medicine, Fu Jen Catholic University, Taipei 24205, Taiwan; 7 Department of Anesthesiology, Cathay General Hospital Sijhih, Taipei 22174, Taiwan; 8 Department of Internal Medicine, Cheng Hsin Rehabilitation Medical Center, Taipei 11220, Taiwan; 9 Department of Medical Research, Cathay General Hospital, Taipei 10630, Taiwan

## Abstract

**Background:**

Chromosome 22q11 deletion syndrome (22q11DS) causes a developmental disorder during the embryonic stage, usually because of hemizygous deletions. The clinical pictures of patients with 22q11DS vary because of polymorphisms: on average, approximately 93% of affected individuals have a de novo deletion of 22q11, and the rest have inherited the same deletion from a parent. Methods using multiple genetic markers are thus important for the accurate detection of these microdeletions.

**Methods:**

We studied 12 babies suspected to carry 22q11DS and 18 age-matched healthy controls from unrelated Taiwanese families. We determined genomic variance using microarray-based comparative genomic hybridization (array-CGH), quantitative real-time polymerase chain reaction (qPCR) and multiplex ligation-dependent probe amplification (MLPA).

**Results:**

Changes in genomic copy number were significantly associated with clinical manifestations for the classical criteria of 22q11DS using MPLA and qPCR (*p *< 0.01). An identical deletion was shown in three affected infants by MLPA. These reduced DNA dosages were also obtained partially using array-CGH and confirmed by qPCR but with some differences in deletion size.

**Conclusion:**

Both MLPA and qPCR could produce a clearly defined range of deleted genomic DNA, whereas there must be a deleted genome that is not distinguishable using MLPA. These data demonstrate that such multiple genetic approaches are necessary for the unambiguous molecular detection of these types of complicated genomic syndromes.

## Background

Chromosome 22q11 deletion syndrome (22q11DS), including DiGeorge syndrome, velocardiofacial syndrome (VCFS) and conotruncal anomaly face syndrome, is the most frequent known chromosomal microdeletion syndrome, with an incidence of 1 in 4000 live births [1]. About 93% of probands have the most common mode, with a de novo deletion of 22q11; 7% have inherited the 22q11 deletion from a parent [2]. However, multiple phenotypic features and associated abnormalities are observed in patients with 22q11DS [3], and phenotypes vary between families because of deletion polymorphisms [4]. In general, congenital heart disease (CHD) is the most common disorder seen, particularly conotruncal malformations [5]. Variable developmental problems and schizoid features are also associated with this syndrome [6].

The molecular basis for 22q11DS is still elusive. The syndrome is apparently caused by a haploinsufficiency of one or more genes that lie in the long arm of chromosome 22 [7]. On average, approximately 90% of affected individuals have a 3 Mb deletion, and 7% have a smaller deletion spanning 1.5 Mb [6]. Fluorescence in situ hybridization (FISH) has proved to be a tool for the detection of 22q11 deletions [8]. However, haploinsufficiency can be confirmed by FISH deletions only when using conventional TUPLE1 or N25 probes, based on assumptions about common deletion breakpoint regions [9,10]. Methods with multiple genetic markers in the 22q11 region are increasingly important for the accurate detection of genomic microdeletions [11,12]. Therefore, microarray-based comparative genomic hybridization (array-CGH) and quantitative real-time PCR (qPCR) have been applied recently in the determination of DNA dosage for 22q11DS [12,13]. Moreover, multiplex ligation-dependent probe amplification (MLPA) analysis with multiple probes has been used for analysing chromosome 22q11 in detail [14]. Many molecular analyses have shown that patients with alterations other than microdeletions in the 22q11 region show features overlapping with 22q11DS [15,16]. These results suggest that this chromosomal region is particularly vulnerable to genomic alterations. Thus, comprehensive molecular evaluation is required to establish the clinical significance of this region.

In this study, MLPA was used to determine genomic DNA dosage in chromosome 22q11 of infants suspected to harbour 22q11DS [14,16]. The range of chromosomal hemizygosity was also explored for genomic microdeletions using array-CGH or qPCR with TaqMan probes to determine the genome changes precisely [13,17].

## Methods

### Participants

Twelve babies suspected to carry 22q11DS (B01 to B12, five boys and seven girls, mean age 2.5 years) from unrelated Taiwanese families were enrolled from the Departments of Pediatrics in Cathay General Hospital and Taipei Medical University Hospital. A family history and an outline classical medical criteria were taken, including testing for hypocalcaemia caused by idiopathic hypoparathyroidism, evaluation for some special forms of CHD such as tetralogy of Fallot with pulmonic stenosis and dysmorphological features typical of the syndrome (Table 1). Another 18 age-matched healthy controls (C01 to C18, 12 boys and six girls, mean age 5.4 years) were collected at Cathay General Hospital. Both patients and the healthy controls were recruited in accordance with the Helsinki Declaration, and met all ethics criteria. Informed consent was obtained from the patients' legal representative before their enrollment. Genomic DNA was obtained from peripheral blood samples of all participants using standard methods [18].

**Table 1:** Clinical characterization of twelve patients suspected to carry 22q11DS

Study subject	Age* at last evaluation	Gender†	Heart defect‡	Dysmorphological facial features	Idiopathic hypocalcaemia	Others
B01	11.6 ys	F	PDA/TR/SVC	asymmetric crying face/high arch palate/bifid uvula	Yes	
B02	4.0 ys	M	PPS	asymmetric crying face/short nasal bridge/flat philtrum/hypertelorism	Yes	
B03	2.5 mo	M	PDA/PPS	bilateral bizarre, low-set ears/micrognathia	nil	*a, b, c*
B04	4.1 ys	M	nil	nil	Yes	*d*
B05	1.9 ys	F	VSD/ASD	short nasal bridge/hypertelorism/thin upper lip	nil	*e*
B06	1.0 mo	F	ECD	cleft palate	nil	*f*
B07	10.4 ys	F	ASD	long, thin nose/cleft palate	nil	
B08	3.5 mo	F	VSD	asymmetric crying face	nil	
B09	7.5 mo	F	nil	iris coloboma	nil	
B10	0.1 mo	F	TOF/PS	nil	nil	
B11	0.2 mo	M	nil	short nasal bridge/hypertelorism/short palpbral fissure/post-rotated and bizarre ears/cleft palate	nil	*a, g, h*
B12	1.0 mo	M	nil	asymmetric crying face/high arch palate	Yes	

*ys = years; mo = months.

† M = male; F = female.

‡ PDA = patent ductus arteriosus; TR = tricuspid regurgitation; SVC = superior vena cava; PPS = peripheral pulmonic stenosis; VSD = ventricular septum defect; ASD = atrial septal defect; ECD = endocardial cushion defect; TOF = tetralogy of Fallot; PS = pulmonic stenosis.

*a*, small penis with hypoplasia of scrotum; *b*, choanal atresia; *c*, trocheo-esophageal fistula; *d*, hypoplastic kidney; *e*, clinodactyly; *f*, left hand polydactyly; *g*, bilateral hand simian crease; *h*, esophageal atresia.

### Multiplex ligation-dependent probe amplification

MLPA with SALSA P023 DiGeorge syndrome/VCFS kits (MRC-Holland, Amsterdam, The Netherlands) using a specifically designed set of probes to detect deletions was performed in all subjects according to the manufacturer's instructions. The ligation products were amplified and analysed as reported previously, with some modification [17]. Briefly, data were normalized against two of four healthy controls (two boys and two girls) in each analysis. DNA dosages with log_2 _ratios below -0.515 were regarded as showing haploinsufficiency [19].

### Array CGH

Aliquots of 50 ng of genomic DNA from the reference normal DNA (Promega, Madison, WI, USA) and experimental samples were amplified using Repli-G Amplification kits (Qiagen, Hilden, Germany) according to the supplier's protocols. Amplified DNA was digested using the restriction endonucleases *Rsa*I and *Alu*I for a minimum of 2 h at 37°C, then verified using DNA 500 chips run on a Bioanalyzer 2100 (Agilent Technologies, Santa Clara, CA, USA). Individual reference and experimental samples were then purified using QIAQuick PCR clean-up kits (Qiagen). Labelling reactions were performed with 10 μg of purified DNA and a Bioprime labelling kit (Invitrogen, Carlsbad, CA, USA) according to the manufacturer's instructions in a volume of 50 μL with a modified dNTP pool containing 120 μM each of dATP, dGTP and dCTP; 60 μM dTTP; and 60 μM Cy5-dUTP (for experimental samples) or Cy3-dUTP (for reference samples; PerkinElmer). Labelled targets were cleaned up using Centricon YM-30 columns (Millipore, Madison, WI, USA). Experimental and reference targets for each hybridization were pooled and mixed in a 500 μL hybridization mixture of 50 μg of human Cot-1 DNA (Invitrogen)/100 μg of yeast tRNA (Invitrogen)/1 × hybridization control target DNA (Agilent Technologies)/1 × hybridization buffer (Agilent Technologies). Before hybridization to the array, the hybridization mixtures were denatured at 95°C for 3 min and incubated at 37°C for 30 min. To remove any precipitate, the mixture was centrifuged at ≥ 14,000 *g *for 5 min, and the supernatant was transferred to a new tube. The labelled and denatured DNA target was then hybridized to a human genome CGH 44A microarray (Agilent Technologies) at 65°C for 40 h. The arrays were then washed in 0.5 × SSC/0.005% Triton X-102 (wash 1) at room temperature for 5 min, followed by 5 min at 37°C in 0.1 × SSC/0.005% Triton X-102 (wash 2). Slides were dried and scanned using an Agilent DNA microarray scanner at 535 nm for Cy3 and at 625 nm for Cy5. Scanned images were analysed using Feature Extraction 8.1 software, and data analysis was performed using CGH Analytics software version 3.2 with a moving average of 2 Mb at a z-score threshold of 2.0 (Agilent Technologies).

### Quantitative real-time PCR

Quantitative real-time PCR was used to quantify the DNA dosage levels for eight probes (BID-2, CO3M, A3M, HIRA-2, M3M, LZTR1-2, T3M and TO3M) in chromosome 22q11 using a LightCycler thermal cycler system, a TaqMan Master kit and a specific probe from the Human Universal Probe Library according to the manufacturer's instructions (Table 2; Roche Diagnostics GmbH, Mannheim, Germany). Each 20 μL reaction contained 50 ng of template DNA and was normalized against a reference endogenous gene (GAPDH, AY340484). Samples retaining both 22q11 alleles were expected to have log_2 _ratios of gene dosage close to zero and log_2 _ratios close to -1 were regarded as indicating low DNA dosage [12,17,20].

**Table 2:** A list of primer and TaqMan probe for quantitative real-time PCR

Genomic location*	Primer Sequence†	Probe number‡
BID-2	F: GTGATCTCGGCTCGCTGTA	10
	R: CAGCTACTGGGGAAGGATTG	
CO3M	F: CAGCATAACCACTGCAGGTC	23
	R: TAAGGAATTGGCTCATGCAA	
A3M	F: GAGCTGCCTACAGCTATCCTG	66
	R: CTGTGCACGTCAGCAACAC	
HIRA-2	F: CATCAGGAAATGCTCTTGGAG	02
	R: GCCGAAGCCTTGAGTTTTAG	
M3M	F: CTGGCTGCACAGGAGACAT	24
	R: GAGGCCTTTCCCTTGTATGC	
LZTR1-2	F: TCCTGTCAGTTTGCCCTTCT	23
	R: TTGCACCACCTAACACTACCA	
T3M	F: GGTCTGCCCAGAATTAGCAC	10
	R: CTGGGTGACTTTCAGCCAAT	
TO3M	F: CTGGAAAATGGGAAGGAACA	25
	R: GCTGCTTCCTCTGCTTGAAA	
GAPDH	F: GCTGCATTCGCCCTCTTA	10
	R: GAGGCTCCTCCAGAATATGTGA	

*BID, NM197966; HIRA, X89887; LZTR1, NM006767; GAPDH, AY340484.

† F = forward primer; R = reverse primer.

‡ Probe number, from the Human Universal Probe Library of Roche Diagnostics GmbH, Mannheim, Germany.

### Statistical analysis

Statistical analysis was carried out using SPSS version 13.0 for Windows (SPSS, Chicago, IL, USA). Fisher's exact test was used to assess the significance of any association between changes in DNA dosage, and the diagnosis of 22q11DS. *P *< 0.05 was considered statistically significant.

## Results

### Distribution of multiple probes on chromosome 22q11

**Figure 1:**
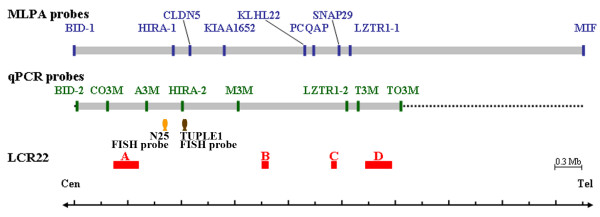
**Relative position of probes and LCR22s on the chromosome 22q11 region**. MLPA probes (blue bars) are arranged according to the manufacturer's instructions (MRC-Holland). qPCR probes (green bars) are designed for each amplicon within and flanking the deletion region. Sequence information for probes CO3M, A3M, M3M, T3M and TO3M, are derived from Chen et al. [20]. Commonly used FISH probes for N25 (a orange fish) TUPLE1 (a brown fish) are indicated. Ranges of LCR22-A, -B, -C, and -D, as defined by Shaikh et al. [39], are depicted as thick red lines. BID-1 and -2, in the genome of BID (NM197966); HIRA-1 and -2, in the genome of HIRA (X89887); LZTR1-1 and -2, in the genome of LZTR1 (NM006767). Genomic distances between probes are proportional to the exact size and size of unit genome is 0.3 Mb as indicated. The orientation of the sequence is centromere (Cen) to telomere (Tel).

The nine MLPA probes hybridize to a 5.7 Mb genomic region on chromosome 22q11, and the eight qPCR probes link to a 3.6 Mb genomic region. Using MLPA, the most commonly deleted region for 22q11DS started at probe HIRA-1 downstream of LCR22-A and ended at probe LZTR1-1 upstream of LCR22-D (Figure 1). The genomic distance between probes HIRA-1 and LZTR1-1 is reduced to 1.70 Mb. Similarly, LCR22-A and LCR22-D flank five probes, A3M to T3M, which are designed in the most deleted region of 22q11DS for the qPCR method. Compared with the MLPA probes, both A3M and T3M are closer to LCR22-A and to LCR22-D (Figure 1).

### Determination of genomic deletions on multiple chromosomes

**Figure 2:**
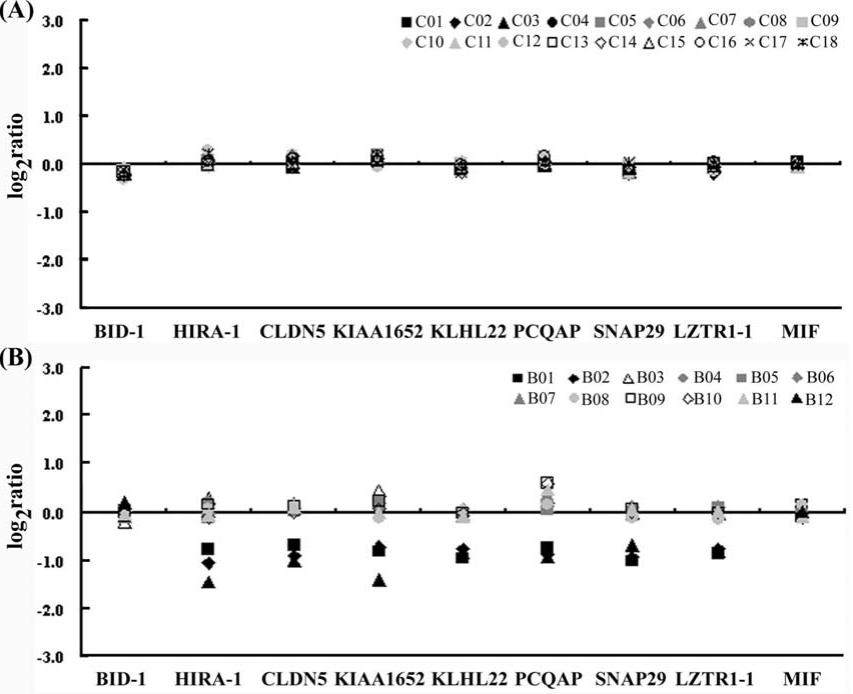
**DNA dosage profiles estimated by MLPA analysis**. Ratios on each DNA dosage are plotted relative to single DNA samples isolated from (A) 18 healthy controls and (B) 12 patients suspected to carry 22q11DS. Nine probes are ordered by position on chromosome 22q.

**Figure 3:**
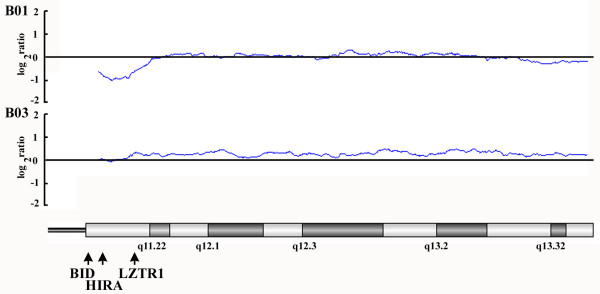
**Variations of DNA dosages on chromosome 22q from two patients suspected to carry 22q11DS by the array-CGH analysis**. Scatter plots delineate the log_2 _ratios of differential signals between probes from experimental samples and reference normal DNA. Positive values indicate DNA dosages of experimental samples being greater than that of reference normal DNA, whereas negative values indicate reduced DNA dosages in experimental samples. The blue line is plotted as the moving average of a window containing the probes within 2 MB. Relative positions of BID, HIRA, and LZTR1 are indicated with black arrows.

MLPA analysis was carried out on blood samples, and profiles of DNA dosages are shown in Figure 2. As shown in Figure 2a, none of the healthy control subjects showed haploinsufficiency for any probes (range of log_2 _ratios, -0.304 to +0.299). In contrast, loss of DNA dosage was found in three of the suspected 22q11DS infants (B01, B02 and B12) on chromosome 22q with an identical deletion (1.70 Mb) from probes HIRA-1 to LZTR1-1 (range of log_2 _ratio, -0.690 to +1.059; Figure 2b). The other nine probands did not show any loss of DNA dosage for any probes on chromosome 22q, and their log_2 _ratios of DNA dosages were all close to zero (range of log_2 _ratio -0.218 to +0.595). DNA dosages for all the probes for other chromosomes assayed (4q, 10p and 8p) showed no changes for the 12 proband infants. However, the changes in genomic copy number in 22q for subjects B01, B02 and B12 were significantly associated with the classical diagnostic criteria for 22q11DS (*P *< 0.01 by Fisher's exact test). MLPA detected a reduced DNA dosage level from subject B01 on chromosome 22q11, and this was also shown by the array-CGH analysis (Figure 3).

### The qPCR method clearly defines genomic changes in chromosome22q11

**Figure 4:**
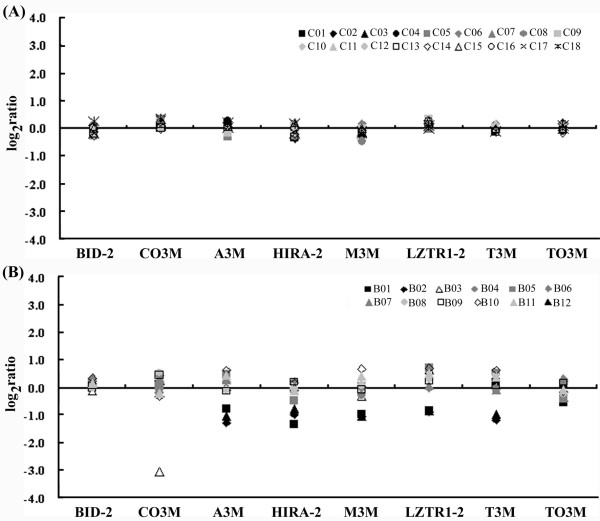
**DNA dosage profiles determined by quantitative real-time PCR**. Histograms of relative DNA dosage for (A) 18 healthy controls and (B) 12 patients suspected to carry 22q11DS. Ratios on each DNA dosage are plotted and normalized with a reference endogenous gene, GAPDH (AY340484). Eight probes are ordered by position on chromosome 22q. Samples retaining both alleles, log_2 _ratio close to 0; samples with deleted one allele, log_2 _ratio close to -1.

To confirm and clearly define genomic deletions in the proband infants, we applied qPCR using TaqMan probes. Compared with the 18 healthy controls, DNA dosage levels from the 12 patients varied independently (Figure 4). Genomic imbalance was detected in four of these babies (B01, B02, B03 and B12), but no detectable changes were found in the other eight babies or in the healthy controls. Changes in genomic copy numbers were also significantly associated with clinical manifestations (*P *< 0.01 by Fisher's exact test). As with the MLPA technique, subjects B01, B02 and B12 showed large genomic deletions in 22q11 by qPCR (range of log_2 _ratios -0.79 to -1.32). However, the size of the deletion in proband B01 was smaller than in B02 and B12. As shown in Figure 4b, an additional haploinsufficiency for probe T3M was detected from probands B02 and B12 (log_2 _ratios -1.15 and -0.95, respectively) but not from subject B01 (log_2 _ratio +0.22). This proband carried a 2.38 Mb deletion (from probes A3M to T3M) whereas subjects B02 and B12 each had a 2.27 Mb deletion (from probes A3M to LZTR1-2). Moreover, subject B03 showed very low DNA dosage (log_2 _ratio -3.06) by qPCR, using probe CO3M.

## Discussion

Copy number variations in some specific genomic regions can lead to genetic disorders [21]. MLPA and qPCR can now be used separately or together to determine genomic copy numbers in many human diseases [17,22,23]. As the clinical features of patients suffering from chromosomal alterations on 22q11 have proved to be extremely polymorphic, different approaches have been applied to determine the DNA copy number in this chromosomal region [20,24]. Reduced dosage of genes within 22q11 is believed to cause the phenotype of 22q11DS [25].

### Molecular basis of chromosome 22q11 from patients with heart malformations

Deletion of chromosome 22q11 appears to be the second most common cause of CHD after Down syndrome [26]. Jiang et al. have used several polymorphic microsatellite markers to determine chromosome 22q11 deletions in patients with isolated CHD [27]. Therefore, genomic markers for these deletions have become the molecular basis for studying heart malformations in patients with 22q11DS. Many studies have compared the FISH approach using commercially available probe (N25 or TUPLE1) to other methods with multiple molecular probes [20,28]. However, the FISH probe detected the typically deleted region around the gene for Histone cell cycle regulation defective, S. cerevisiae, homolog A (HIRA; X89887) (Figure 1) [10]. Atypical deletions and deletion polymorphisms won't be detected by this conventional approach [29-32]. In this study, we used three approaches – MLPA, array-CGH and qPCR – with multiple molecular markers to detect genomic copy numbers in chromosome 22q11 for these 12 babies with CHD, idiopathic hypocalcaemia, or dysmorphological facial features. MLPA has been used frequently to detect duplications and deletions in 22q11 [16,28,33,34], and qPCR with universal TaqMan probes was first specifically used in the determination of DNA dosage for subjects with 22q11DS. We found that both MLPA and qPCR were rapid, reliable, cost-effective, high-throughput methods for diagnosing 22q11DS with statistical significance. In our analysis, MLPA and qPCR produced almost the same measure of haploinsufficiency for chromosome 22q11 of three of the probands (B01, B02 and B12) using probes LCR22-A to LCR22-D, as reported by others using different methods [12,20].

### Different chromosomal deletions on chromosome 22q11 from patients

We confirmed the presence of the 3 Mb common deletion that accounts for 90% of patients with 22q11DS [6]. Probands B01, B02 and B12 had the same degree of DNA deletion between probes HIRA-1 and LZTR1-1 using the MLPA method. Nevertheless, the fragment size detected by qPCR (2.38 Mb or 2.27 Mb) was larger than that detected by MLPA (1.70 Mb). This difference resulted from the different distributions of probes in these two methods. Briefly, all three of these subjects showed genomic deletions starting from probe A3M, which hybridizes about 0.36 Mb upstream from probe HIRA-1 on 22q. In addition, both B02 and B12 showed haploinsufficiency that extended to probe T3M, which binds downstream of probe LZTR1-1. The deleted genome of probands B02 and B012 on chromosome 22q11 is at least about 105 Kb longer than when measured using MLPA. This deleted region detected specifically by qPCR contains at least four known genes, including SLC7A4 (NW927495), which are associated with VCFS when deleted [35].

### Complex genomic conditions in patients with 22q11DS or in suspected cases

Clinically, two of the three haploinsufficient probands were diagnosed with CHD (B01 with patent ductus arteriosus and B02 with peripheral pulmonic stenosis). Five of the other babies with CHD, including ventricular septum defect, atrial septal defect, endocardial cushion defect, tetralogy of Fallot, and pulmonic stenosis, did not show any genomic deletions within the region of LCR22-A to -D using MLPA or qPCR. Considering these results along with previous reports, we agree that two or more types of deletions are close in size and position within the 3 Mb common deletion [20]. We also noted that two of the proband infants with atrial septal defect were without detectable deletions in this region, as reported by others [27]. However, one (B03) presented very low DNA dosage measured by qPCR using the CO3M probe from two independent analyses. The male baby was born with gestational age 34 weeks, birth body weight 1886 gm, body length 47 cm, and head circumference 29 cm via Cesarean section. He has no family history of chromosomal anomaly, but presents with the clinical manifestations of triangle face, bilateral malformation of ears without external meatus, markedly low-set ears, micrognathia, small penis with hypoplasia of scrotum, and choanal atresia (Table 1). There still have many works to evaluate if any single nucleotide polymorphism or mutation exist within the region of CO3M and to correlate this bias of DNA dosage with the clinical manifestations of subject B03. However, we speculate that such complex genomic conditions might be common in patients with 22q11DS or in suspected cases.

### MLPA and qPCR overmatch the complex, expensive and time-consuming methods for detecting genomic microdeletions

CGH has been used to screen for multiple chromosomal aberrations, and it is now frequently united with microarray hybridization for assessing the level of any chromosomal imbalance [36,37]. Based on phenotypes resembling that of 22q11DS, two suspected patients were examined with array-CGH for whole genome defects [4,38]. However, no chromosomal imbalance was detected, with the exception of chromosome 22q. This result agreed with that obtained by MLPA to some degree, because only the probes on chromosome 22q represent genomic deletions whereas probes on chromosomes 4q, 10p and 8p indicate normal copy numbers. On the other hand, the implementation of qPCR detection in clinical laboratories will address the need to replace complex, expensive and time-consuming methods for detecting genomic microdeletions or duplications of clinical importance [12].

## Conclusion

In conclusion, both MLPA and qPCR produced clearly defined ranges of deleted genomic DNA. These two molecular diagnostic methods are quick, easily manipulated and complementary. For the purpose of molecular diagnosis without any aberration, multiple molecular approaches are necessary for a complicated genomic syndrome such as 22q11DS. Further studies on the comprehensive genomic profiling of subjects with 22q11DS will help the symptomatic treatment and prenatal diagnosis of fetuses with affected siblings.

## Competing interests

The authors declare that they have no competing interests.

## Authors' contributions

CY and CHH designed the study, collected the samples and drafted the manuscript. MLC participated in the design of the study. KLH and LHL collected the samples and control individuals that they characterized. YSY and CCC participated in analyzing the data and performing the statistical analyses. HCH helped to draft the manuscript. CWC performed the MLPA analysis. CJH conceived the study, participated in its design and coordination, carried out the molecular genetic studies and helped to draft the manuscript. All authors read and approved the final manuscript.

## Pre-publication history

The pre-publication history for this paper can be accessed here:


